# Joint issues – conflicts of interest, the ASR hip and suggestions for managing surgical conflicts of interest

**DOI:** 10.1186/1472-6939-15-63

**Published:** 2014-08-15

**Authors:** Jane Johnson, Wendy Rogers

**Affiliations:** 1 Department of Philosophy, Macquarie University, Sydney, NSW 2109, Australia; 2 Department of Philosophy and Australian School of Advanced Medicine, Macquarie University, Sydney, NSW 2109, Australia

**Keywords:** ASR hip, Conflicts of interest, Disclosure, Recusal, Surgical innovation

## Abstract

**Background:**

Financial and nonfinancial conflicts of interest in medicine and surgery are troubling because they have the capacity to skew decision making in ways that might be detrimental to patient care and well-being. The recent case of the Articular Surface Replacement (ASR) hip provides a vivid illustration of the harmful effects of conflicts of interest in surgery.

**Discussion:**

We identify financial and nonfinancial conflicts of interest experienced by surgeons, hospitals and regulators in the ASR case. These conflicts may have impacted surgical advice, decision-making and evidence gathering with respect to the ASR prosthesis, and contributed to the significant harms experienced by patients in whom the hip was implanted. Drawing on this case we explore shortcomings in the standard responses to conflicts of interest – disclosure and recusal. We argue disclosure is necessary but by no means sufficient to address conflicts of interest. Using the concept of recusal we develop remedies including second opinions and third party consent which may be effective in mitigating conflicts, but their implementation introduces new challenges.

**Summary:**

Deployment of the ASR hip is a case of surgical innovation gone wrong. As we show, there were multiple conflicts of interest involved in the introduction of the ASR hip into practice and subsequent attempts to gloss over the mounting body of evidence about its lack of safety and effectiveness. Conflicts of interest in surgery are often not well managed. We suggest strategies in this paper which can minimise the conflicts of interest associated with surgical innovation.

## Background

Surgical innovation raises ethical concerns to do with increased risk of harms to patients, inadequate informed consent, conflicts of interest and potentially inappropriate health care spending. The recent case of the Articular Surface Replacement (ASR) hip provides an excellent illustration of the ways these ethical concerns are not idle philosophical speculation, but manifest and play out in practice. In this paper we focus solely on conflicts of interest regarding surgical innovation. We identify a number of financial and other conflicts implicated in the case and examine how their effects might be prevented or at least mitigated in the future.

The paper begins by outlining the conflicts of interest that were involved, before briefly describing salient features of the ASR case and identifying the financial and nonfinancial conflicts that arose with respect to this prosthesis. Finally, we consider remedies to help prevent or mitigate some of the harmful effects that conflicts of interest have the potential to generate in surgery.

Conflicts of interest in surgery, and in healthcare generally, are of concern because they have the capacity to skew decision making in ways that might possibly, but not necessarily, impact patient care adversely. Broadly speaking, these conflicts can arise in professional contexts when a secondary interest has the potential to distract from and/or affect a primary one [1,2]. Primary interests are those directly related to fulfilling a particular role and in the case of surgeons, these include acting in the best interests of patients, promoting patient welfare, performing surgery only in areas they are competent to do so and so forth. Conversely, secondary interests are not directly related to the role in question, and might include receiving a payment for designing a device or promoting a particular product. Such secondary interests may be entirely legitimate; however they remain conflicts and have the potential to distract from primary interests or inappropriately bias the professional in their primary capacity.

With respect to surgeons, the bias caused by conflicts of interest can influence decision making in one of two ways. First, decisions might be affected quite deliberately with a surgeon intentionally choosing a particular device because of a financial relationship with the manufacturer, such as being a consultant for them. Such purposeful actions are unprofessional, unethical, and potentially fraudulent. Second, a surgeon might be influenced unconsciously; for instance, being unaware that their reason for a preferring a particular device derives from a favourable relationship with a manufacturer. There is evidence that unconscious bias is potentially more widespread and harmful than conscious bias (which may be considered a form of bribery). This is because the decision-maker is unaware of it, and the bias can arise from apparently innocuous interactions and gifts, which are known to trigger feelings of reciprocity in the beneficiary [3].

There is also evidence that surgeons are more tolerant of conflicts of interest than, for instance, medical physicians [4]. This is arguably the result of changes in the relationships between surgeons and device manufacturers over time such that industry representatives are frequently now part of the theatre team, regarded as colleagues and friends [4]. Contemporary innovations tend to be driven by industry rather than surgeons, with the former seeking out prominent surgeons to be involved in what can sometimes amount to expensive ‘me too’ devices with unclear or unnecessary indications [5].

The nature and extent of financial relationships between industry and surgeons are hard to track. However, work by Hockenberry et al. reveals payments from five device manufacturers to orthopaedic surgeons disclosed as part of the settlement of a US Department of Justice law suit. The payments were for consulting, royalties and research support. In 2007, the mean payment received by orthopaedic surgeons from the five manufactures was $190,331. In 2010, it was $233,108 (based on data available from three firms only). Some payments were in excess of $1,000,000 [6].

Financial conflicts of interest receive the most attention, where the secondary interest competing with the primary one derives from some type of monetary incentive or reward. As we discuss below, however, nonfinancial conflicts also arise in surgery and may be more challenging to identify and remedy.

As well as impacting on the decision making of surgeons, conflicts of interest can also influence patient decision making, and the way healthcare funding is disbursed.

The recent case of the Articular Surface Replacement (ASR) hip provides a vivid illustration of the effects of conflicts of interest.

DePuy Orthopedics (a subsidiary of Johnson and Johnson) launched the ASR hip in the late 1990s. The hip used metal-on-metal technology with components made from cobalt, chromium and titanium. There were two versions of the ASR prosthesis– one offered total hip replacement^a^ and the other the resurfacing of natural hips.^b^ ASR hips involved a smaller surgical incision than competitors [7] and the metal-on-metal technology promised reduced risk of fracture and dislocation, good positional sensitivity, and greater longevity [8]. Compared to total hip replacement, the resurfacing option offered quicker recovery rates and the potential for greater and lasting mobility; these features opened up the possibility that younger and more active individuals (who require a robust and long-lasting hip replacement) could be candidates for the ASR prosthesis.

Both types of ASR hip were approved for use in the European Union and Australia, while in the United States only the total hip replacement was approved, though US surgeons could use the resurfacing option ‘off label’ [9].

Over time ASR hips have been implicated in a range of localised and systemic health problems including pain, inflammation, infection, tiredness, nausea, visual impairments, problems with mobility, tinnitus, heart palpitations and depression [10]. Perhaps the most unequivocal measure of the failure of both versions of the ASR hip is their high revision rate (i.e. the need to re-operate). These problems seem to have arisen primarily as a result of the design of the ASR hip which can lead to metal grinding on metal. This in turn contributes to disintegration of the prosthesis, leakage of cobalt and chromium ions and subsequent toxicity.

In 2010 DePuy, after a protracted campaign denying that there were problems with the prostheses, issued a worldwide recall of the ASR hip.

In the following section we identify a range of conflicts of interest that arose in the case of the ASR hip and suggest ways in which such conflicts and their impact might be prevented, or at least mitigated, in the future.

## Discussion

### Financial conflicts of interest and the ASR hip

The ASR case involved financial conflicts of interest on the part of surgeons, hospitals and regulators and these played out in ways that arguably contributed to patient harm.

Surgeons involved in the design of the ASR hip prostheses received significant royalties, which, if properly managed, could be consistent with ethical and professional norms. Legislation in the US requires disclosure of relevant company payments so that it is known that in 2009–10 designer Thomas Schmalzried received a little under US $3 million and Thomas Vail just over US $500,000 [9]. Apart from legitimate royalties, DePuy provided direct financial inducements to orthopaedic surgeons to use their products through the use of sham consulting agreements [11]. In addition, some surgeons were recruited as speakers for industry-sponsored and industry-funded events aimed at other surgeons. Some idea of the scale of these activities may be gleaned from fines levied on DePuy. For example, in the US DePuy paid $84.7 million to avoid prosecution over inducements it paid to surgeons to use DePuy products [10], while in the UK the company was fined almost £5 million for unlawful payments to doctors in Greece [9]. These financial incentives had the capacity to influence surgical advice and decision making in favour of the ASR hip.

Informed consent may have been compromised when surgeons failed to notify patients of the financial benefits they stood to gain upon use of the ASR prosthesis. Patients may have considered this information relevant to their decision as to whether to proceed with this surgeon, this operation and this particular prosthesis. There is evidence that informed consent was compromised in at least two cases involving Roger Oakeshott, one of the six designers of the ASR hip. Two of Oakeshott’s patients expressed surprise when told, after experiencing problems with the hip, that Oakeshott had been involved in its design [7].

Another potential impact of surgeons’ conflicts of interest involves the collection and presentation of evidence about the performance of the prosthesis. In this instance rather than reporting problems with the ASR device, some surgeons with links to DePuy simply stopped using the hip [9]. This may have prevented these surgeons from inflicting further patient harm, but is ethically inadequate as a response since it hindered evidence gathering. A more appropriate response would have involved reporting the problems which led these surgeons to abandon the prosthesis, which may in turn have lead to an earlier recall of the hip, with fewer patients exposed to the device.

There is some controversy over whether the problems that emerged in relation to the ASR hip could have been predicted [9]. Whether or not this is the case, there is evidence that material produced by surgeons with financial links to DePuy sought to discount emerging concerns about the prosthesis. For instance, in response to high revision rates identified through the Australian National Joint Replacement Registry, Professor Vail, an ASR design surgeon, wrote a ‘white paper’ indicating how the Australian data ought to be interpreted. The paper explained away the Australian results by suggesting they did not properly factor in learning curves^c^ and that Australian results differed from those obtained in other jurisdictions [9]. Another designer, Thomas Schmalzried, wrote a paper ‘Setting the record straight on metal hypersensitivity’ intended to allay concerns regarding the metal-on-metal technology employed in the ASR hips [12]. Although there can be genuine disagreement over facts and their interpretation in medical science, thus opening up a legitimate place for academic debate about the merits of particular procedures or devices, we now know that the position taken in these papers did not adequately reflect other evidence available to DePuy.

Surgeons were not the only group with financial conflicts of interest over the ASR hip. Hospitals and other health care providers received financial incentives to use the device in their institutions. For example in the US Johnson and Johnson were fined for making “improper payments to publicly employed health care providers in Greece, Poland and Romania in order to induce the purchase of medical devices and pharmaceuticals” produced by their subsidiaries such as DePuy [9], p. 3. In Australia, a Senate Committee heard from a patient who claimed that Johnson and Johnson’s sponsorship of a hospital fellowship was linked to the hospital’s use of the company’s products [10].

Regulatory bodies were likewise not immune from conflicts of interest related to these prostheses. In the UK the Medicines and Healthcare Products Regulatory Agency established an expert advisory group to investigate the risks of metal-on-metal technology. Three of the eight members had conflicts of interest; two were consultants to DePuy and one worked for Smith and Nephew, the company which produced the competitor metal-on-metal Birmingham hip [12].

### Nonfinancial conflicts of interest in the ASR case

As well as financial conflicts, it is reasonable to assume that the ASR case involved non-financial conflicts for surgeons, both external as well as within role. As we discuss below, nonfinancial conflicts may be difficult to identify and substantiate in particular cases, and be controversial in a way that financial conflicts are not. It may therefore seem both odd and problematic to make reference to such conflicts. However there are at least three reasons to do so. First, it appears that non-financial conflicts are frequently overlooked partly because financial ones are more familiar, more straightforward to identify and seemingly easier to manage or potentially resolve. Ease of identification and redress are not, however, compelling ethical reasons to focus solely on financial conflicts. Second, non-financial conflicts can influence behaviour even though they may be difficult to identify and their precise impact hard to assess. Research suggests patients do not understand that non-financial conflicts, such as generated by being an industry consultant or speaker, or designer of a prosthesis, can bias surgeons [13]. Third, these conflicts need to be raised and discussed in order to develop adequate and comprehensive responses to conflicts of interest in surgery.

Nonfinancial external conflicts of interest have as their secondary interest an incentive or motivation which conflicts with a surgeon’s primary role with respect to providing care for the patient. These secondary interests may be legitimate in their own right, but risk compromising patient care when they come into conflict with a primary interest. Morreim et al. identifies a mix of interests which may motivate surgical innovation, including secondary and within role ones, such as:

‘‘the intangible reward of emotional excitement from breaking new ground and making new discoveries, the possibility of enhanced reputation and of academic advancement through discovery of new information and its publication; the possibility of obtaining grants, awards, or contracts to create a formal clinical trial if an innovation is successful, and perhaps the satisfaction of very strong personal dedication to advancing the science of medicine’’ [14], p. 1957.

As it is not possible to know or measure an individual’s psychological state or motivations, it is equally not possible to identify accurately the specific nonfinancial rewards surgeon innovators in the ASR case may have experienced. Nonetheless it is important to at least consider that such nonfinancial rewards may have played a role in this case and note that we should aim to develop ways in which the adverse impacts of such rewards may be minimized in the future.

Within role conflicts arise in surgery because two legitimate *primary* surgical obligations - to act in the patient’s best interests, and to innovate for the benefit of current and future patients^d^ can be in tension such that pursuing one can compromise the other. In seeking to innovate, an individual patient’s care might be compromised by the surgeon’s unsubstantiated optimism about the innovation’s advantages, superiority over alternatives, their own skill in performing that innovation, favourable complication rates and so forth. Such conflicts are problematic because surgery necessarily involves uncertainty and questions of judgement, thus opening up a space in which unconscious bias can inappropriately influence patient care [5].

Given the conflicts of interest that emerge from the ASR case, we now consider possible remedies which might prevent or at least mitigate conflicts and their injurious impacts in the future.^e^

### Possible remedies

#### Financial conflicts

Conflicts of interest may be addressed by preventative measures such as avoidance, and measures to address existing conflicts such as disclosure, divestment, and recusal [2,15]. How effective might these have been in the ASR case? In considering these traditional remedies we canvass other possible remedies, some of which, as we note, are more feasible to implement than others.

Disclosure about the financial rewards dispensed by companies like device manufacturers to surgeons and hospitals is increasing. The existence of laws such as the Sunshine Act in the US means, for instance, that we know the sums involved in the royalty payments to the US based ASR designers and in fact all monies paid to physicians and surgeons by industry. However, while disclosure may reveal financial conflicts of interest, in and of itself disclosure does nothing to remedy them or mitigate their potential biasing effects.^f^ Disclosure may in fact give the appearance that conflicts of interest are being dealt with in a way that is not consonant with the actual impact of disclosure.^g^ This view is supported by a 2013 survey which found that 71% of surgeons agreed that simply declaring monies received for speaking, consulting, travel or research is adequate for dealing with financial conflicts of interest [4].

We agree with authors [16,17] who note that disclosure does not address the effects of conflicts of interest but rather shifts the burden onto the recipient of that information, such as patients. However, disclosure does serve some purposes, such as identifying the presence of financial ties, and opening these up to scrutiny as to their potential justifiability. For instance, while it may be reasonable for surgeons to receive royalty payments for their role in developing or designing devices^h^, other forms of payment may lack comparable justification. Payments for simply using a product or achieving certain quotas are ethically indefensible, as are payments where a role is being remunerated which appears tokenistic, for example in some cases where surgeons act as consultants or spokespeople. In this instance disclosure helps draw attention to the full range of remunerations and thus can identify payments that appear to be little more than direct incentives to use specific products. Once identified, professional or institutional policy should make rulings on which kinds of payments are acceptable and why, and prohibit those that are aimed at promotion or marketing. We think that disclosure is a warranted and feasible first step in addressing conflicts of interest.

How disclosure is achieved also matters. Simply having information available on the public record about a surgeon’s role in developing a device and any financial payments they may receive as a result of designing or using that device is insufficient, as research indicates that less than one third of patients would look at a website disclosing financial ties [16]. It seems likely that disclosure to patients by the individual surgeon is essential to fully informed consent.^i^ Again, however, being transparent about a conflict does not necessarily dilute its power to influence decision making, and as social science research suggests, may paradoxically increase bias [18]. The surgeon, having been open about their financial conflict, may then, intentionally or otherwise, present a more partial view of the evidence for using the device [19]. The effect of disclosure on patients is unpredictable. In response to learning of the surgeon’s conflict, the patient may be wary of or discount the surgeon’s advice; or they may respond favourably to this disclosure and value what might be perceived as the surgeon’s honesty. They might even regard it as admirable that their surgeon has shown confidence in a device through investing in its development [18].

Likewise in the case of hospitals and other health care providers, disclosure about industry payments appears necessary but insufficient to mitigate the possible effects wrought by conflicts of interest. However, disclosure of the nature of interactions and magnitude of payments may signal a need to question their justification. For instance, depending on the context and the manner in which it is undertaken, it may be appropriate for hospitals to use industry funding for training their surgeons in the use of new devices. Surgeons need to be trained if they are to competently and effectively use innovative products and it may be preferable to have this training based at a local hospital rather than provided to individual surgeons in more exotic locations. Payments that raise greater concerns include those which attempt to induce hospitals to purchase a particular device or which provide financial incentives to use specific devices or meet quotas. As with individual surgeons, disclosure is a necessary first step in identifying and assessing the nature of the interactions leading to payment. The next step is the development of policy to manage justifiable interactions and prohibit those that cannot be justified on the basis of enhancing patient care. Again, this is a suggestion which we argue could be successfully implemented.

Thus transparency^j^ on its own appears to be insufficient to address the conflicts of interest raised by the ASR case. Disclosure can however help determine when divestment of the interest (for example severing a conflicted relationship, or selling relevant shares) or recusal is appropriate, and assist with compliance checking. We now turn to the strategy of recusal, which is necessary if the conflict cannot be resolved by divestment of the interest.

Recusal requires an individual to step back from decision making when they have a conflict of interest to ensure the conflict does not inappropriately influence decision making. In the case of the ASR hip this would mean requiring that those surgeons who have designed the hip or received other financial payments from DePuy or Johnson and Johnson, be excluded from the process of recommending the hip to patients, though they might still be permitted to perform the relevant surgery if recommended by others. However this suggestion seems problematic on a number of fronts, and might, contra the intent of recusal, impact adversely on patient care. Part of a surgeon’s role is diagnostic and involves assessing a patient’s suitability for a particular procedure, in addition to then performing that procedure. It seems likely that a surgeon experienced with a particular device or procedure might be better positioned to make the appropriate judgements to ensure the best outcomes for a patient.^k^ This may however be compromised by the optimism bias we discuss below.

Perhaps rather than recusal then, a financially conflicted surgeon might be required to recommend that patients receive a second opinion from a surgeon who is not similarly conflicted.^l^ But this strategy is also problematic. Such a measure assumes the second surgeon is neutral and does not have his/her own conflict with respect to other manufacturers or devices. If the surgical environment is highly competitive, a surgeon might be inclined to recommend their favoured option regardless of patient circumstances. Alternately, a surgical environment with a culture of collegiality might lead the second surgeon to simply backup the recommendation of the first so as not to undermine professional authority. Seeking a second opinion also takes time and incurs additional costs, and unless mandated or widely accepted, might create mistrust between the patient and surgeon. If the surgeon him or herself recommends that the patient seek a second opinion, the patient may be concerned that the surgeon lacks faith in their own skills and judgement. Conversely, if initiated by the patient, the surgeon may feel mistrusted by the patient. A further issue is that offering patients the choice of surgeon – the provider of standard treatment, or the surgeon who prefers device A, or B, or C etc. may place too heavy a burden on individual patients to circumvent conflicts of interest. A patient, already vulnerable, may be left feeling bewildered and disempowered when confronted with a variety of advice offered by experts in an area outside their ken. Thus a system of second opinions is not at this point in time a practical means of addressing surgical conflicts of interest.

Another way of dealing with this situation would be for a surgeon to recuse themselves from the procedures surrounding consent; rather than the treating surgeon explaining and describing the procedure, a third party could be charged with undertaking this process. This would enable a person without the relevant conflict to provide information including the number of times the surgeon has performed a procedure; how the procedure or device differs from the standard version; the risks (insofar as they can be anticipated); the state of evidence about safety and efficacy etc. This process would also allow a patient to reach a decision about for example, whether to opt for the ASR or Birmingham hip, away from the direct sphere of influence of their surgeon. This would potentially allow patients to act in line with their risk profile – some will be keen to be the first to have a new procedure, while others may be more risk averse [17]. Again, however, this strategy has shortcomings. In the first instance a surgeon has a moral obligation to ensure a patient’s consent is genuinely informed and freely given so that transferring responsibility for consent onto a third party could be regarded as failing to fulfil this obligation.^m^ Second, unless this process was normalized it would, as in the case of second opinions, have the potential to undermine the surgeon-patient relationship. Third, there would be issues over who could provide this neutral consent process, and how additional costs and logistical issues should be managed. Thus while this suggestion too presents significant practical challenges, features of it such as determining the amount of information to be provided to patients in situations of conflicts of interest could be implemented. For example, institutional committees could mandate the use of coloured consent forms in cases of potential conflicts of interest, and this would prompt the surgeon to disclose and discuss their conflict.

Another feasible strategy for managing the conflicts of interest of institutions, hospital administrators, managers and others charged with making purchasing decisions about devices and equipment is for these individuals to simply recuse themselves from decisions in situations where they or the organization have benefited financially from manufacturers.

Recusal also seems an appropriate and manageable response to conflicts of interest on the part of those making regulatory decisions. Thus the composition of the UK’s expert advisory group on metal-on-metal technology should not have included individuals linked to the ASR or Birmingham hips. If the kind of knowledge and expertise possessed by these individuals was required to inform the process (as seems possible), it could have been sought from these individuals qua expert advisors with no direct role in making decisions and recommendations.

Developing strategies other than disclosure, divestment and recusal are important given some of the shortcomings in these mechanisms noted above, as well as the apparent necessity of industry-surgeon links to facilitate device innovation,^n^ and the ostensible tolerance of surgeons to conflicts of interest discussed earlier. An option suggested by Paul Thagard is to increase individuals’ awareness of how the brain functions when making ethical decisions. As he comments “[p]erhaps if people knew more about how cognition and affect are intimately connected and how the connections are inaccessible to conscious introspection, they would be much less confident about the basis and validity of their decisions” [20], p. 378. Surgical education and continuing professional development could incorporate relevant work in moral neuropsychology explaining the ways in which conflicts of interest lead to unconscious bias. This might have the effect of de-stigmatising conflicts of interest for surgeons while simultaneously forcing them to take seriously mechanisms to minimize their untoward impacts. Educational initiatives could teach surgeons about the neuropsychological basis of the bias that results from conflicts of interest. This would help them to appreciate that being impacted by conflicts of interest (at least unconsciously) is not an individual moral failing; and that avoidance is the most reliable way to avoid the effects of conflicts of interest. Such open discussion might encourage surgeons as a professional group to develop stronger policies to avoid and manage conflicts.

### Nonfinancial and within role conflicts

Some of the suggestions outlined above to deal with financial conflicts (e.g. third party consent, second opinions etc.) also have the potential to address nonfinancial and within role conflicts. Inserting a neutral barrier between the conflicted surgeon and their patient, may mediate the conflict in some cases. Likewise disclosure is a necessary first step in dealing with non-financial conflicts.^o^ We note that avoidance and divestment are less likely to be applicable to non-financial and within role conflicts, as we do not advocate surgeons withdrawing from research and teaching.

Another way in which nonfinancial and within role conflicts might be addressed in cases such as the ASR hip is through mandating the collection of relevant evidence for or against the innovation and disseminating this information.^p^ The bias engendered by conflicts might be tempered in the face of evidence which contradicts the surgeon’s optimistic view of the innovation. This could be achieved via compulsory registration of innovations such as the ASR hip^q^ so that outcomes data on safety and efficacy are collected and made available in an independent and objective fashion. This is not a new suggestion [21-23], and in an earlier paper we flagged its potential to address conflicts of interest [24]. We also noted two challenges in implementing this approach, to do with defining and identifying innovation (which is by no means straightforward [25] and in this case would depend on surgeons self identifying innovation); and in funding such a mechanism.^r^

The gathering, analysis and dissemination of emerging evidence have the potential to reduce patient harms, bolster confidence in surgery and generate cost savings in publically resourced health systems. In our view, this is a strong argument to fund such ventures, possibly through pioneering new funding models.^s^ As we have suggested, it might also be possible to collect data through local or institutional registers of innovative procedures and their outcomes, or by developing existing review structures such as new interventions committees. Such committees could function in a similar way to institutional review boards established to review research, setting out conditions for informed consent (which might include mandatory third party provision of information) and monitoring the introduction of innovations by collecting data on outcomes. And while mandating national or international systems of registration is challenging, it is within the remit of health care institutions to develop their own systems for monitoring innovations within their premises and using this information to better inform both patients and practitioners.

Our suggested remedies to reduce the incidence and effects of conflicts of interest in surgery are summarized below.

At a minimum, we suggest:

1. Disclosure at an institutional level of all gifts, payments and interactions between individual surgeons and industry, with self-disclosure triangulated against mandated industry disclosure of recipients of payments, and significant penalties (e.g. loss of practising privileges) for failure to comply;

2. Disclosure by institutions of their remunerated interactions with industry;

3. Review of the justifiability of 1 and 2, for example by a formally constituted conflicts of interest review committee, all of whose members lack relevant conflicts, and which is charged with developing policy for points 4 and 5;

4. Development of institutional policy on the acceptability of relevant activities and the prohibition of those that do not contribute to improving patient care (for example, activities that promote sales of specific devices);

5. Development of workable procedures for informing the local community about: the nature and effects of conflicts of interest; and the steps undertaken at the institution for managing conflicts, including provision of second opinions or brokered consent;

6. Recusal of any conflicted employees of the institution from decision-making about relevant matters;

7. Development of local and national procedures for collecting, analysing and disseminating data on surgical innovations;

8. Development of mandatory modules on understanding the neuropsychology of conflicts of interest in surgical education and continuing education programs.

## Summary

The implementation of the ASR hip is a case of surgical innovation gone wrong. Though the hip promised to address problems with earlier devices and to extend the benefits of hip replacement to a new cohort of patients, it failed to deliver on this promise and instead resulted in significant patient harms. As we have shown, there were multiple conflicts of interest involved in the uptake of the ASR hip as well as attempts to gloss over the mounting body of evidence about its lack of safety and efficacy.

Conflicts of interest in surgery are not currently well managed, with the traditional mechanism of disclosure and recusal harbouring significant limitations.

Conflicts of interest need to be taken seriously in order to avoid harms such as those experienced by recipients of the ASR hip. We hope the strategies we have sketched out in this paper can begin a process of engaging surgeons, regulators, hospitals, patients and others in avoiding the avoidable and minimising the unavoidable conflicts of interest that are associated with surgical innovation.

### Ethics

Ethics approval was not required for this paper.

### Endnotes

^a^This version involves removing and replacing the head of the thighbone (the femoral head) and the damaged socket (acetabulum) with metal.

^b^Resurfacing technology is newer. In the case of the ASR hip it involves capping or resurfacing the femoral head with a hemispherical covering, while a shell is used to replace damaged bone and cartilage within the socket.

^c^The term ‘learning curve’ is used to refer to the heightened risks to patients incurred while an individual surgeon or surgical team obtains competency in procedures that are new to them.

^d^We have argued elsewhere that pursuing innovation is a primary surgical obligation, i.e. that engaging in at least some innovation (such as devising a new hip prosthesis) is a valid and even essential part of what it is to be a good surgeon. W. Rogers and J. Johnson: **Addressing Within-Role Conflicts of Interest in Surgery**, *Journal of Bioethical Inquiry* 2013; **10**: 219–225.

^e^It is worth noting that some of the behaviour associated with this case may have been fraudulent or illegal; these are criminal matters and our suggestions do not deal with these issues.

^f^It may be, however, that disclosure can act as a prompt or mechanism for reflection on the part of those who are required to disclose payments, relationships etc. Steinbrook R: **Industry Payments to Physicians: Lessons from Orthopedic Surgery.***Arch Intern Med.* 2011; **171(19):** 1765–66.

^g^In their study Lieberman et.al. found that patients had a poor understanding of financial conflicts of interest in spite of having detailed discussions on the issue. JR. Lieberman, MJ. Pensak, MS. Kelleher, RR. Leger, and GG. Polkowski: **Disclosure of Financial Conflicts of Interest: An Evaluation of Orthopaedic Surgery patients’ Understanding.***Clin Orthop Relat Res* 2013; **471**: 472–477.

^h^This is consistent with findings by Camp et. al. indicating that patients were more concerned by gifts given to surgeons by industry (perceived to benefit surgeons and industry) than by payments, such as royalties, regarded as being for activities associated with patient benefit. We also note their suggestion that surgeons in receipt of royalties should donate these when using the device in question for their own patients.

^i^According to research by Lieberman et. al. regarding financial conflicts of interest, supplying written information to patients at an office visit is insufficient to ensure patient comprehension. JR. Lieberman, MJ. Pensak, MS. Kelleher, RR. Leger, and GG. Polkowski: **Disclosure of Financial Conflicts of Interest: An Evaluation of Orthopaedic Surgery patients’ Understanding.***Clin Orthop Relat Res* 2013; **471**: 472–477. However, we note the findings of Camp et. al. that less than 50% of patients want to discuss the surgeon’s financial relationships in the pre-operative consultation.

^j^We note other problems with transparency including the difficulties of: defining the scope of disclosure (especially as insignificant gifts can create bias); mandating reporting; ensuring compliance (including the need for triangulation through disclosure of industry payments to individuals); and enforcing penalties. Kesselheim AS and Maisel WH: **Managing Financial and Nonfinancial Conflicts of Interest in Healthcare Delivery.***American J of Therapeutics* 2010, **17**: 440–443.

^k^It seems patients at least appear to believe that surgeons involved in the design of prosthesis will have a greater clinical expertise and understanding of the device and that this will likely lead to better outcomes. J.R. Lieberman, M.J. Pensak, M.S. Kelleher, R.R. Leger, and G.G. Polkowski. **Disclosure of Financial Conflicts of Interest: An Evaluation of Orthopaedic Surgery patients’ Understanding.***Clin Orthop Relat Res* 2013; **471**: 472–477.

^l^This section builds on an earlier paper focused on nonfinancial conflicts where we first raised these issues. W. Rogers and J. Johnson **Addressing Within-Role Conflicts of Interest in Surgery**, *Journal of Bioethical Inquiry 2013;***10**: 219–225. Kesselheim and Maisel also suggest that second opinions may be warranted in some circumstances. Kesselheim AS and Maisel WH: **Managing Financial and Nonfinancial Conflicts of Interest in Healthcare Delivery.***American J of Therapeutics* 2010, **17**: 440–443.

^m^This could be circumvented by a patient returning to their surgeon after the third party consent process so that the surgeon can ensure the patient has fully understood what the procedure involves and agreed to that procedure.

^n^Camp et. al. claim banning financial relationships between industry and surgeons will not work as surgical input is essential to successful innovation. McKneally also makes a case for the importance of industry partnerships to surgical innovation, maintaining that, properly managed, they can describe a virtuous rather than a vicious cycle. MF McKneally: **Beyond Disclosure: Managing Conflicts of Interest to Strengthen Trust in Our Profession**. *Journal of thoracic and Cardiovascular Surgery* 2007; **133**: 300–2, 301. DiPaula and colleagues refer to American’s peak body for orthopaedic surgeons’ consensus statement which notes enhanced patient care requires collaboration with industry. DiPaola CP, Dea N, Noonan VK, Bailey CS, Dvorak MFS, Fisher CG: **Surgeon-industry conflict of interest: survey of North American’s opinions regarding surgeons consulting with industry**. *The Spine J* 2013, doi: 10.1016/j.spinee.2013.06.028.

^o^See for instance the discussion in Kesselheim and Maisel, p. 442. Kesselheim AS and Maisel WH: **Managing Financial and Nonfinancial Conflicts of Interest in Healthcare Delivery.***American J of Therapeutics* 2010, **17**: 440–443.

^p^Surgical research is poorly funded in comparison to pharmaceutical research. There is however more research involving surgical devices and products than research into techniques and procedures.

^q^The role of the Australian National Joint Replacement Registry in drawing attention to the ASR hip by identifying high revision rates, was noted earlier.

^r^Registers which collect and collate information in a manner that is useful and relevant for patients are expensive to establish and maintain.

^s^For instance, in order to secure a funding base to support independent clinical research, the Italian Parliament introduced a law requiring pharmaceutical companies to pay a fee amounting to 5% of their marketing budget (excluding salaries). Maybe device manufacturers could be required to contribute to a similar scheme to support the development of registers. Garattini S and Bertele V: **Comparative clinical effectiveness**. *Eurohealth* 2009; **15**, 4–6.

## Competing interests

The authors declare that they have no competing interests.

## Authors’ contributions

JJ and WR developed the conceptual analysis. JJ wrote the first draft, incorporating edits and suggestions on this draft from WR. JJ and WR revised and edited a number of drafts. JJ and WR read and approved the final manuscript.

## Pre-publication history

The pre-publication history for this paper can be accessed here:

http://www.biomedcentral.com/1472-6939/15/63/prepub
